# *Cis*-regulatory elements operating in the trophoblast

**DOI:** 10.3389/fcell.2025.1661952

**Published:** 2025-11-24

**Authors:** Terezia Vcelkova, Paulina A. Latos

**Affiliations:** Center for Anatomy and Cell Biology, Medical University of Vienna, Vienna, Austria

**Keywords:** trophoblast, placenta, silencers, enhancers, gene regulation

## Abstract

The placenta is vital for supporting embryonic development and ensuring a successful pregnancy. Its diverse functions are carried out by specialized trophoblast cell types, including the progenitor cytotrophoblast, the multinucleated syncytiotrophoblast, and the invasive extravillous trophoblast. The distinct identities of these cells are governed by tightly regulated gene expression programs, controlled by transcription factors and cis-regulatory elements, particularly enhancers and silencers. They integrate spatiotemporal cues to modulate transcriptional activity and establish cell-type-specific gene expression profiles. Disruptions of these regulatory mechanisms can impair placental development and function, contributing to pregnancy complications. In this review, we explore the interplay between TFs and CREs in trophoblast lineage specification and function, with a focus on enhancers and silencers. We provide an overview of human placental development, describe commonly used *in vitro* models, and discuss recent technological advances that have deepened our understanding of transcriptional regulation in the placenta.

## Introduction

The placenta sustains embryonic development and a successful pregnancy. It provides the site of exchange for nutrients, gases, and metabolites between the mother and the embryo. It also acts as an endocrine organ, producing and secreting hormones that regulate pregnancy adaptations and have immunoprotective roles. To fulfill the diverse functions, the placenta contains a range of highly specialized trophoblast cell types. For instance, the multinucleated syncytiotrophoblast (STB) that constitutes the actual site of exchange between the maternal and fetal bloodstreams, the highly invasive extravillous trophoblast (EVT) then invades the uterus and remodels maternal arteries, and the cytotrophoblast (CTB), the progenitor population that gives rise to both the STB and the EVT (Velicky et al., 2016; Turco and Moffett, 2019). The distinct identities of these cell types are determined by their unique gene expression programs. The cell-specific transcriptional outputs result from integrating the spatio-temporal information, including signaling cues, and are largely controlled by *cis*-regulatory elements (Frost et al., 2023; Yu et al., 2023). These DNA elements can be functionally divided into promoters, enhancers, silencers, and insulators. Promoters are the 0.1–1 kb sequences close to the transcriptional start site (TSS) of a gene, harbor multiple binding sites for transcription factors (TFs), and recruit RNA polymerase for transcriptional initiation. Basal transcription can be further regulated by the distally located enhancers, silencers, and insulators. Enhancers carry a unique chromatin signature and were originally defined as sequences that activate transcription independent of orientation and direction. In contrast, silencers repress the transcription. Consequently, the deletion of an enhancer leads to transcriptional downregulation of its target gene(s), while the removal of a silencer leads to upregulation. Insulators are genetic elements that protect genes from genomic position effects, e.g., block enhancers or silencers from interacting with the promoter (Kim and Wysocka, 2023). Studying cis-regulatory elements—particularly enhancers and silencers—is essential for understanding the molecular mechanisms that control gene expression during placental development. Proper regulation of these elements is critical for trophoblast differentiation and invasion, key processes in establishing a functional placenta. Disruption of these regulatory pathways has been linked to major placental disorders, including preeclampsia, intrauterine growth restriction, gestational trophoblastic disease, placenta accreta spectrum, and gestational diabetes mellitus, all of which contribute significantly to maternal and fetal morbidity and mortality worldwide (Wilkerson and Ogunbodede, 2019; Wang et al., 2020; Keighley et al., 2024).

In this review, we focus on the cis-regulatory elements, in particular enhancers and silencers, operating in the trophoblast during placental development. We provide an overview of human placental development, including the trophoblast cell types that contribute to its formation, and describe commonly used *in vitro* models. Furthermore, we discuss how the cis-regulatory elements control transcription and present the recent advancements in our understanding of how these elements determine trophoblast cell identities and drive placental development and disease.

## Placental development

**FIGURE 1:**
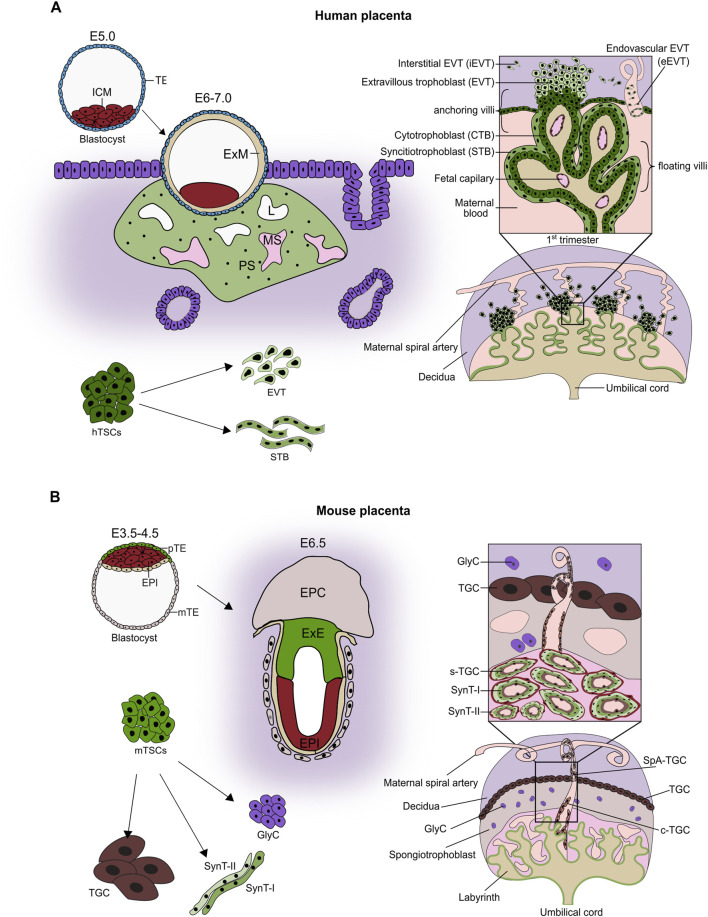
Placental development. **(A)** Around 4–5 days post-fertilization, the blastocyst forms with an inner cell mass (ICM) and trophectoderm. Following implantation (E6.0–7.0), cytotrophoblast (CTB) fuses to form a primitive syncytium (PS). The lacunae (L) appear within PS and connect to maternal capillaries, forming maternal sinusoids (MS). CTB projections through the PS initiate primary villi formation, followed by the secondary villi, and leading to the development of the villous tree by the end of the first trimester. By this time, the placenta is composed of villous trees with mesenchymal cores containing fetal capillaries and three trophoblast lineages: the progenitor population of cytotrophoblast (CTB), the syncytiotrophoblast (STB), and the extravillous trophoblast (EVT). In the floating villi, which are immersed in maternal blood, a layer of mononuclear CTB is overlain by a multinucleated STB layer formed by the continuous fusion of CTB. In contrast, anchoring villi that attach to the decidua contain CTB columns that serve as progenitor zones for invasive EVT. EVT differentiates into endovascular EVT (eEVT), which remodels maternal spiral arteries, and interstitial EVT (iEVT), which infiltrates the decidua. Human trophoblast stem cells (hTSCs) represent the proliferative CTB progenitor population. They are self-renewing and bi-potent, i.e., capable of differentiating into both STB and EVT. **(B)** In the mouse, shortly after implantation, the mural TE differentiates into polyploid primary parietal trophoblast giant cells (pP-TGCs). The polar TE (overlaying the ICM) proliferates, powered by Fgf4 secreted by ICM, and gives rise to the extraembryonic ectoderm (ExE) and the ectoplacental cone (EPC). ExE develops further into the chorion, which later fuses with the allantois and triggers differentiation of the trophoblast cells into two layers of multinucleated syncytiotrophoblast cells (SynT-I and SynT-II) through cell-cell fusion, and a layer of mononucleated sinusoid TGCs (S-TGCs) that line the maternal blood vessels. The EPC differentiates into a spongiotrophoblast layer containing spongiotrophoblast cells (SpT), Glycogen cells (GlyC), and several types of TGCs, including spiral artery-associated TGCs (SpA-TGCs) and canal TGCs (C-TGCs). The established placenta comprises the labyrinth, the junctional zone (made up of spongiotrophoblast and glycogen cells), and the outermost layer of parietal TGCs adjacent to maternal decidua. Within the labyrinth, the exchange barrier consists of three trophoblast cell types: S-TGCs, SynT-I, SynT-II surrounded by fetal vasculature. The self-renewing and multipotent mouse trophoblast stem cells (mTSCs) can be derived from TE, ExE, and parts of the chorion (Figure adapted from (Papuchova and Latos, 2022)).

After fertilization, the human embryo undergoes several rounds of cleavages and forms a morula. The outer blastomeres differentiate into the trophectoderm (TE) of the blastocyst, whereas the inner ones become the inner cell mass (ICM). Later in development, the TE will give rise to the trophoblast part of the placenta, while the ICM will give rise to all embryonic derivatives. Around 6–7 days post-fertilization (dpf), the blastocyst implants into the endometrial lining of the uterus (Hamilton and Boyd, 1966). The TE gives rise to the mononuclear cytotrophoblast (CTB) that fuses and forms a multinuclear primary syncytium (PS), which invades the endometrium (Figure 1A). The invasive PS establishes a system of vacuoles that fuse into lacunar spaces, breach the maternal capillaries, and give rise to the maternal blood sinusoids. The PS also erodes endometrial glands, ensuring access of the developing embryo to the nutrient-rich uterine secretions. Concomitantly, the CTB underneath the PS intensely proliferates and forms projections that push through the PS, creating primary villi. The primary villi are thus made of the CTB core overlaid by the multinucleated syncytium. The CTB projections proliferate further and merge laterally, forming a cytotrophoblast shell, while the space between them becomes the intervillous space. Subsequently, the villous core is invaded by extraembryonic mesoderm (ExM), presumably originating from the ICM, resulting in the formation of the secondary villi. The tertiary villi are established with the development of the fetal blood capillaries in the villous core around 18 dpf. Additional proliferation, differentiation, and branching events result in the formation of the villous trees (Figure 1A) (Hertig et al., 1956).

The fully established placenta is formed by extensively branched villous trees. The villous tree contains anchoring villi, which mount the placenta to the uterus, and the free-floating villi that bathe in the maternal blood. In the anchoring villi, resulting from CTB proliferation, a cell column forms that attaches to the uterine wall (Schlafke and Enders, 1975). The proximal part of the column contains proliferative progenitor cells that give rise to the highly invasive and migratory EVT lineage. They are divided into interstitial (iEVT) and endovascular (eEVT). iEVTs invade and migrate within the decidua (endometrium during pregnancy) between the vasculature, whereas eEVTs invade into the blood vessels, replace endothelial cells, and adopt a pseudo-endothelial phenotype. They remodel the maternal spiral arteries, increasing the blood flow volume and decreasing the shear stress damage to uterine vessels. In addition, at the early stages of placental development, the eEVTs move down the arteries and form plugs that prevent blood flow and ensure a hypoxic environment. These plugs disintegrate by the end of the first trimester (Pijnenborg et al., 1980; Burton et al., 1999).

The floating villi consist of the mesenchymal villous core, covered by the two layers of trophoblast cells: mononuclear CTB and multinuclear STB. The proliferative CTB serves as a progenitor population that both maintains itself and contributes cells upon differentiation and fusion to the STB. The STB is the outermost layer, remains in direct contact with maternal blood, and forms a large, multinucleate syncytium (Moffett and Loke, 2006). It provides the actual site of exchange of gases, nutrients, and metabolites between the maternal and fetal bloodstreams, and acts as a vital endocrine organ by producing and secreting pregnancy-related hormones. To facilitate the function, the STB surface is covered by a brush of microvilli, which enlarges the exchange surface and is highly enriched in a plethora of transporters and receptors (Robinson et al., 2009). STB also plays immunoprotective roles. On one hand, STB does not express the human leukocyte antigen (HLA), protecting the placenta and the embryo from recognition as foreign by the maternal immune system. On the other hand, STB facilitates transport of maternal immunoglobulin G to the fetus via expression of the neonatal Fc receptor (Simister et al., 1996).

Despite having corresponding functions, the human and murine placenta exhibit striking morphological and molecular differences. Up to the blastocyst stage, the development progresses similarly in both species. After implantation of the murine embryo, the polar (overlaying the ICM) TE proliferates, giving rise to the extraembryonic ectoderm (ExE) and the ectoplacental cone (EPC). The ExE develops further into a chorion, which later fuses with the allantois- an outgrowth derived from the extraembryonic mesoderm, itself a derivative of ICM. This chorioallantoic fusion, taking place around E8.5, enables invasion of the ExM-derived blood vessels and triggers folding and differentiation of chorion into the three trophoblast lineages: syncytiotrophoblast I, syncytiotrophoblast II, and sinusoidal giant cells lining the maternal blood sinuses. Together with the concomitant branching morphogenesis, it leads to the establishment of the labyrinth zone of the murine placenta, which facilitates maternal-fetal exchange and is functionally related to the human chorionic villi. In addition to the labyrinth zone, the murine placenta contains the junctional zone that develops from the EPC (Hemberger et al., 2020). It comprises the secondary parietal giant cells (TGCs), the spongiotrophoblast, and the glycogen cells (GlyCs), which serve as an energy storage (Figure 1B). The TGCs invade the decidua and remodel maternal spiral arteries to ensure optimal blood flow, and thus functionally correspond to the human EVTs. Together, while the general functional layout of the human and murine placenta is similar, the morphological and molecular differences do exist.

Recent years have seen unprecedented progress in understanding the molecular basis of the human placental function. These advances have been driven by the rapid development of -omics technologies, and the establishment of novel, reliable *in vitro* models of human trophoblast (Haider et al., 2018; Okae et al., 2018; Turco et al., 2018; Sheridan et al., 2020; Hori et al., 2024). The high-throughput transcriptomic, chromatin, and proteomic analysis of placental tissues from different stages of gestation, often performed at the single-cell resolution, provided us with a comprehensive atlas of cell states during placental development (Table 1) (Liu et al., 2018; Vento-Tormo et al., 2018; Castel et al., 2020; Khan et al., 2021; Zhang et al., 2021; Wang et al., 2022; Wang et al.,2024; Shannon et al., 2024; Uhm et al., 2025). Another layer of information was acquired by the spatial transcriptomics analysis of the placental tissue across gestation, where the gene expression data were complemented by the spatial information, offering genuine snapshots of placental development (Table 1) (Arutyunyan et al., 2023; Bhalla et al., 2023; Greenbaum et al., 2023; Ounadjela et al., 2024). While the study of the primary tissue offers a valuable blueprint, access to such material has been severely limited, hindering the development of reliable *in vitro* models and functional assays until recently. For years, researchers primarily relied on transformed or cancer-derived cell lines with limited potential, as well as animal models, mainly murine. The establishment of the self-renewing human trophoblast stem cell (TSC) and self-organizing trophoblast organoid (TO) models thus marked a breakthrough. The TSCs correspond to the CTB progenitor population of the first trimester placenta. Under culture conditions that activate EGF and WNT signaling and inhibit TGF-ß and ROCK pathways, TSCs self-renew and retain bipotency. Upon induction in lineage-specific media, they differentiate into multinucleated STB and invasive EVT, representing the three major trophoblast lineages of the developing placenta (Okae et al., 2018). Under similar conditions and supported by Matrigel, TOs self-organize in 3D into outer CTB-like and inner STB-like compartments, recapitulating the inside-out architecture of human villi (Haider et al., 2018; Turco et al., 2018). Another approach to study earlier stages of placental development relies on the use of human naive pluripotent stem cells (hnPSCs). They represent human pre-implantation epiblast and are consistent with the late epiblast and trophectoderm cell fate determination observed in human blastocyst and can give rise to trophoblast stem cells, providing a useful tool to study trophoblast specification and differentiation (Castel et al., 2020; Dong et al., 2020; Dong et al.,2022; Io et al., 2021). Further advances led to the generation of a human blastocyst model termed blastoids from hnPSCs by harnessing their capacity to differentiate into both embryonic (epiblast) and extraembryonic (trophectoderm and hypoblast) lineages. The blastoids faithfully recapitulate the early human blastocysts in terms of size, structure, cell types, transcriptome, epigenome, and ability to develop to post-implantation stages, offering another model to study the earliest stages of trophoblast development in the relevant biological context (Sozen et al., 2021; Kagawa et al., 2022; Karvas et al., 2023). Together, the stem cell- and organoid-based trophoblast models are developmentally relevant, scalable, amenable to genetic modifications, suitable for genetic and chemical screens, and as such, offer reliable tools to study molecular mechanisms driving trophoblast development and disease. Indeed, numerous recent studies have combined these *in vitro* models with genetic manipulations, high-throughput screens, various -omics approaches and tissue referencing to uncover novel regulators and dissected their function during placental development (Haider et al., 2018; Dong et al., 2020; Dong et al.,2022; Saha et al., 2020; Varberg et al., 2021; Varberg et al., 2023; Chen et al., 2022; Karvas et al., 2022; Ray et al., 2022; Arutyunyan et al., 2023; Dietrich et al., 2023; Li et al., 2024; Lea et al., 2025).

**TABLE 1:** Summary of single-cell and single-nucleus RNA-seq studies and key findings in human trophoblast.

Study	Approach	Material	Findings
Liu et al. (2018) Cell Res.	scRNA-seq	Full-term placental trophoblast cells	Identified multiple trophoblast subtypes and differentiation trajectories.
Vento-Tormo et al. (2018) Nature	scRNA-seq	1st-trimester decidua and placenta	Charted diverse maternal and fetal cell types and unveiled ligand–receptor interaction networks underpinning immune tolerance.
Castel et al. (2020) Cell Rep.	scRNA-seq	1st trimester vCTBs and EVTs hTSCs, hiTSCs, hcTSCs	h(i/c) TSCs are akin to day 8 trophoblasts of the human embryo.
Zhang et al. (2021) Mol Genet Genomic Med.	scRNA-seq	Normal (35-36 week) and preeclamptic (38-39 week) placentas	The vCTBs and EVTs were involved in immune responses, and endoplasmic reticulum signaling was upregulated in STBs in preeclamptic placentas.
Khan et al. (2021) Front Cell Dev Biol	scRNA-seq	ESC-derived in vitro trophoblast lineages	BAP-treated ESCs revealed diverse trophoblast lineages, including distinct STB subtypes, with oxygen levels shaping their gene expression profiles.
Wang et al. (2022) Sci Rep.	scRNA-seq	Full-term maternal–fetal interface	Found trophoblast progenitor‑like cells and implicated PRDM6 in EVT differentiation.
Arutyunyan et al. (2023) Nature	snRNA-seq, snATAC-seq, spatial transcriptomics	Sections of 1st trimester implantation site	Spatially resolved multiomic single-cell atlas of the maternal–fetal interface, including the myometrium; defined the transcriptomes of invasive trophoblast: placental bed giant cells and endovascular EVTs; model the dual role of interstitial EVTs and endovascular EVTs.
Greenbaum et al. (2023) Nature	spatial transcriptomics and proteomics	66 pregnancies between 6 and 20 weeks of gestation	Detailed spatio-temporal map of early placental development; insights into how immune cells, EVTs and arteries coordinate during spiral artery remodeling.
Bhalla et al. (2023) Placenta	spatial transcriptomics	Normal and preeclamptic term placentas	Distinct RNA patterns associated with morphology and preeclampsia.
Wang et al. (2024) Nat Genet.	snRNA-seq, snATAC-seq	101,500 nuclei from 12 placentas from (6-9 weeks) to (38-39 weeks)	Comprehensive single-nucleus multiomic map of human STB nuclei across gestation, mapping their developmental heterogeneity, functional specialization, and associated transcriptional networks.
Shannon et al. (2024) Dev Cell.	scRNA-seq	Primary trophoblast and stem cell-derived trophoblast organoids	Organoids largely mimic key placental cell types, but lack some mature subtypes, highlighting both their utility and limitations as placental models.
Ounadjela et al. (2024) Nat Med.	snRNA-seq, snATAC-seq, spatial transcriptomic	1st-trimester placenta; 1 million cells across weeks 6-11 of gestation	Mapped 17 major cell types, reconstructed developmental trajectories, identified regulatory elements, and TFs, found tumor-like and immune evasion gene programs, mapped cell-cell communication pathways.
Uhm et al. (2025) Cell Biosci.	snRNA-seq	Fetal membrane samples from spontaneous preterm birth and controls	Spontaneous preterm birth subtypes have distinct placental cell compositions, gene expression patterns, and cell signaling pathways.

Summary of single-cell and single-nucleus studies investigating human trophoblast, highlighting key findings on cell populations and regulatory programs, as well as the tissue sources analyzed.

## Enhancers

**FIGURE 2:**
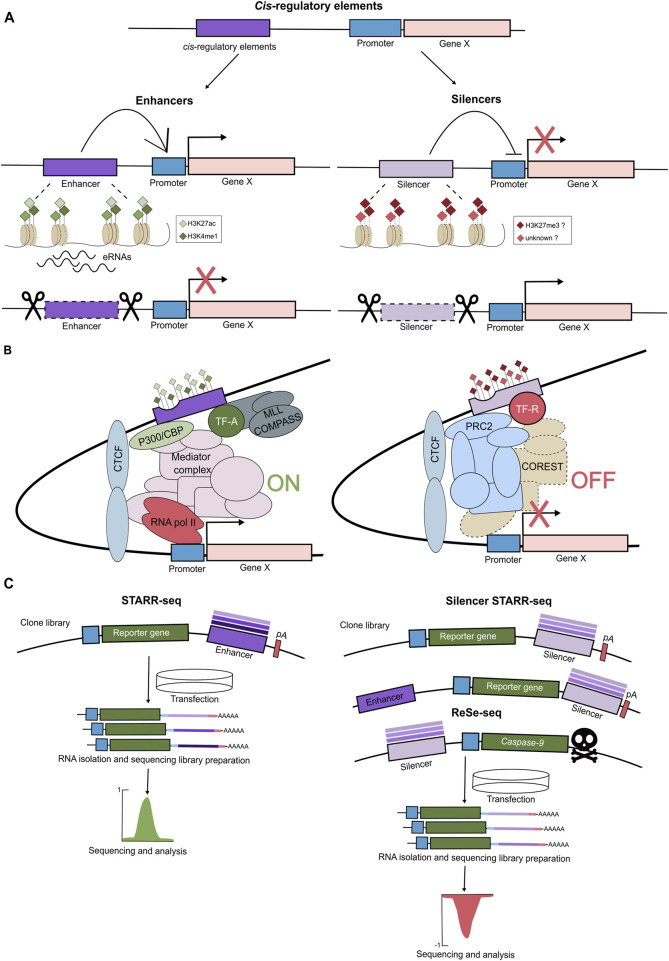
The cis-regulatory elements: enhancers and silencers. **(A)** The *cis*-regulatory elements (CREs) are regions of non-coding DNA regulating transcription of neighbouring genes and involve enhancers and silencers. Enhancers activate/increase expression of the target genes and are marked by open chromatin, activating histone marks (H3K4me1, H3K27ac) and transcription of enhancer RNAs (eRNAs). When removed, the expression of their target gene is reduced. Silencers suppress/decrease transcription of the target genes, and their removal leads to gene activation. Silencers tend to exhibit open chromatin and, at least some classes, are associated with H3K27me3, though their chromatin features remain less defined. **(B)** Enhancers and silencers regulate gene expression within chromatin loops by interacting with promoters. Enhancers contain multiple TF binding motifs, bind activating TFs (TF-A) and recruit histone acetyltransferase p300/CBP that deposits acetyl marks, MLL/COMPASS complex that monomethylates H3K4, and together they promote transcription via Mediator and RNAPolII complexes. Silencers recruit repressive TF (TF-R), PRC2/H3K27me3, and repressive complexes (e.g., COREST), leading to transcriptional repression. **(C)** High-throughput assays such as STARR-seq, ss-STARR-seq, and ReSE enable functional identification of enhancer and silencer elements genome-wide, based on their effect on reporter gene expression. Self-Transcribing Active Regulatory Region sequencing (STARR-seq) has been developed to identify enhancers genome-wide. In this method, randomly sheared genomic DNA fragments (approx. 600 bp) are cloned downstream of a reporter gene within a plasmid vector. The reporter gene is driven by a minimal promoter followed by a polyadenylation signal (pA). This plasmid library is transfected into target cells, after which total RNA is isolated. Both the reporter RNA and the input DNA library are sequenced. If a genomic fragment functions as an enhancer, it will drive transcription of the reporter gene, resulting in a high RNA to DNA ratio-indicating enhancer activity. Recently, STARR-seq has been adapted to identify silencers (ss-STARR-seq). Randomly fragmented genomic DNA is cloned either downstream or upstream of the reporter gene. To better mimic the native genomic environment, the constructs may also include constitutively active enhancers. Repressive elements reduce the expression of the reporter gene, and thus, silencer activity is inferred from a decreased RNA-to-DNA ratio, regardless of enhancer presence. Additionally, a method that measures the repressive ability of silencer elements (Repressive Element Screening - ReSE) has been employed to assess the silencing potential of genomic sequences. In this assay, DNA fragments are inserted upstream of an inducible gene encoding caspase 9, a protein triggering apoptosis. Upon gene induction, cells containing non-repressive elements undergo apoptosis, whereas cells harboring effective silencers survive. Surviving cells are collected, and the repressive DNA fragments are identified via next-generation sequencing.

Enhancers entered the stage over 40 years ago, with reports that short sequences within the SV40 viral DNA can enhance transcription from a minimal promoter independent of their direction and distance (Treisman and Maniatis, 1985; Zenke et al., 1986). These and subsequent findings led to the original definition of an enhancer as a DNA sequence that activates transcription independent of orientation and direction. Importantly, enhancers are enriched in binding motifs for a variety of TFs and characterized by a unique chromatin constitution. They exist in the open chromatin configuration and contain nucleosome-free regions, as demonstrated by the MNase-seq, DNase-seq, and ATAC-seq approaches. Another hallmark of enhancers is their massive enrichment in the H3K27ac and H3K4me1 histone modifications as well as in binding of the histone acetyltransferase P300 and the Mediator complex (Figure 2A) (Nair et al., 2022; Thomas and Buecker, 2023). The Mediator complex is a large, multi-subunit coactivator complex. It interacts with TFs and co-factors, facilitates the assembly of the preinitiation complex at core promoters, and regulates RNA polymerase II activity (Richter et al., 2022; Ramasamy et al., 2023). In addition, enhancers themselves are transcribed, giving rise to unstable, non-coding eRNAs that are functionally important for enhancer activity (Ntini and Marsico, 2019). Clusters of enhancers that drive expression of genes essential for cell identity are often referred to as super-enhancers (SEs) (Kagey et al., 2010; Whyte et al., 2013). They are characterized by unusually high composite density of cell-type-specific TFs, increased occupancy of the Mediator complex, and exhibit elevated levels of active histone modification, including H3K27ac (Figure 2B). SEs act as crucial regulatory hubs orchestrating key gene expression programs. Interestingly, their elements can act in a redundant, additive, or super-additive fashion (Ma et al., 2024). While identification of putative enhancers based on epigenetic mapping is relatively straightforward, it should be complemented by functional assays, demonstrating their ability to boost the expression of target genes. Alternative approaches to identify enhancers in a high-throughput manner rely on their ability to activate reporter genes and are referred to as massively parallel reporter assays (MPRAs). Libraries of DNA sequences are cloned into a transiently transfected reporter plasmid or a reporter locus in the genome and assayed for their capacity to boost expression. The reported DNA library sequences represent the entire genomes (Arnold et al., 2013; Muerdter et al., 2018; Peng et al., 2020), a selected subset (Barakat et al., 2018), and artificially designed sequences (de Almeida et al., 2022). For instance, in the STARR-seq approach, the reporter plasmid contains the minimal promoter, the reporter gene (e.g., luciferase), and the downstream polyadenylation (polyA) signal. The tested potential enhancer sequences are cloned between the reporter gene and the polyadenylation (polyA) signal. The luciferase expression serves as a readout of enhancer activity, with a predefined cut-off used to distinguish between intrinsic and enhancer-driven expression (Figure 2C) (Arnold et al., 2013). While MPRAs are powerful high-throughput methods for identifying novel enhancers, they present several limitations. First, they are typically performed in a specific cell type and may fail to detect enhancers that are active in other developmental or cellular contexts. Second, the choice of promoter is critical, as recent studies suggest that enhancers can exhibit promoter specificity, activating some promoters but not others. For instance, in *Drosophila*, housekeeping promoters are preferentially activated by housekeeping enhancers, while developmental enhancers typically activate developmental promoters (Arnold et al., 2013; Zabidi et al., 2015; Loubiere et al., 2024). Moreover, the MPRA readouts are quantified on the pass-the-threshold approach, raising the possibility that some weaker enhancers remain undetected. This limitation is especially relevant in light of recent findings showing that many elements with canonical enhancer chromatin signatures are only functional when acting cooperatively with other enhancers, and not in isolation (Thomas et al., 2021; Blayney et al., 2023). Therefore, the most reliable, though labor-intensive, method to validate enhancer function remains its deletion at the endogenous genomic locus, within the native chromatin environment, and, in the appropriate developmental context, to assess its impact on expression of neighboring target genes. Alternatively, a more scalable approach involves enhancer silencing via targeted heterochromatin induction. This can be achieved using a catalytically dead Cas9 fused to the KRAB repressor domain, in combination with a gRNA library, enabling high-throughput silencing of candidate enhancers (Fulco et al., 2016). Overall, a combination of complementary experimental strategies is essential for the robust identification and functional characterization of enhancers across diverse cellular and developmental contexts.

Enhancer-promoter (E-P) communication over long genomic distances is primarily mediated by chromatin looping, a mechanism uncovered by the chromatin conformation capture (3C) technologies. These methods involve crosslinking chromatin, enzymatic digestion, and ligation of DNA fragments that are spatially proximate. Sequencing of the resulting products reveals which regions interact in 3D nuclear space. These studies have shown that the genome is organized into topologically associated domains (TADs)- self-interacting regions within which E-P contacts are enriched (Szabo et al., 2019). TAD boundaries are usually marked by the CCCTC-binding factor (CTCF) and the cohesin complex, both of which contribute to loop formation. Strong interactions at TAD borders facilitate the structural compartmentalization of chromatin, with most E-P interactions occurring within the same TAD (Szabo et al., 2019). E-P interactions exhibit complex architectures, including one-to-many and many-to-many looping patterns (Jiang et al., 2016; Allahyar et al., 2018). Several molecular players are involved in driving these loops. For instance, LIM domain-binding protein 1 (LDB1) mediates looping via homodimerization (Krivega et al., 2014). Other key regulators include YY1, CTCF, cohesin complex, Mediator complex and regulatory RNAs (Kagey et al., 2010; Weintraub et al., 2017; Hansen et al., 2019). Enhancers are thought to regulate transcription by controlling gene bursting kinetics either by increasing the burst frequency or burst amplitude (Fukaya et al., 2016; Larsson et al., 2019).

Often during development and cell differentiation, the shutdown of cell-type-specific programs requires silencing of active enhancers, a process known as enhancer decommissioning. It is usually initiated by loss of key TFs, leading to active removal of H3K27ac by HDACs (Wang et al., 2009), and in some cases LSD1-mediated removal of H3K4me1 (Whyte et al., 2012). Additionally, repressive TFs can replace active ones to further inactivate enhancers. Taken together, enhancers are key cis-regulatory DNA elements controlling gene expression, often acting over long genomic distances. They function in a cell-type-specific manner and play essential roles in development and differentiation. Although characterized by distinctive chromatin features, enhancers require functional validation in relevant biological contexts. With improvements in high-throughput single-cell technologies, we advance our understanding of enhancer activity, specificity, and their contributions to gene regulatory networks.

## Silencers

Silencers are defined as cis-acting, position- and orientation-independent elements that orchestrate active transcriptional repression of target gene(s). They are enriched in binding sites for repressors, a subclass of TFs that negatively regulate gene expression. Despite their vital role in controlling developmental gene expression patterns, particularly in switching off inappropriate genetic programs, silencers are severely understudied. In contrast to enhancers, silencers lack a distinctive chromatin signature that would facilitate their high-throughput identification and subsequent validation. However, they do exhibit relatively open, accessible chromatin, as indicated by the ATAC-seq and DNase I hypersensitivity assays. In addition, it has been reported that silencers are enriched in H3K27me3, but these findings have been debated and likely refer to one subclass of silencers (Figures 2A,B) (Shibata and Crawford, 2009; Hansen et al., 2019; Huang et al., 2019).

One of the best-studied silencers in the human genome is the neuron-restrictive silencer element, also referred to as repressor element 1 (RE1), initially found associated with ∼2,000 neuronal genes. It contains a 21-bp motif recognized and bound by the RE1 silencing transcription factor (REST), which in turn recruits co-factors like the Co-REST complex to repress expression of neuronal genes in non-neuronal and undifferentiated tissues. The Co-REST complex harbors chromatin-modifying activities, histone deacetylases 1 and 2 (HDAC1/2), and histone lysine demethylase 1 (KDM1, also known as LSD1) that facilitate transcriptional repression (Andrés et al., 1999; Bruce et al., 2004; Roopra et al., 2004).

The systematic identification and characterization of silencers lags far behind that of enhancers, though the experimental approaches are similar and include high-throughput screens and computational predictions. Jayavelu et al., tested ∼7,500 selected elements that exhibited open chromatin (as measured by DNase I accessibility) and were devoid of H3K4me3, H3K4me1, and CTCF-binding for their silencer activity using a massively parallel reporter assay (MPRA). Using the MPRA analysis, the authors trained a support vector machine (SVM) classifier and predicted more than 1.7 million candidate silencer elements in the human genome and ∼1 million in the mouse genome, across 82 and 22 cell types, respectively. They were enriched in motifs for known (e.g., BACH2, JUN, NRF1) and RFX repressors. Moreover, HiC data revealed that over 50% of silencers interacted with gene promoters showing no or low expression (Doni Jayavelu et al., 2020). Similarly, chromatin accessibility was exploited in the repressive ability of silencer elements (ReSE) screen (Figure 2C). In this approach, accessible regions from K562 and HepG2 cell lines were screened for their ability to repress cell death by silencing the transcription of an inducible caspase 9 reporter gene. While ∼2,600 and ∼1,660 silencers were identified in K562 and HepG2, respectively, only ∼2% were shared between the 2 cell types, indicating high cell specificity of silencers (Pang and Snyder, 2020).

In addition to accessible chromatin, another predictor, for at least a subclass of silencers, was enrichment in H3K27me3. These regions, referred to as the H3K27me-rich regions (MRRs), preferentially interacted with each other and their target genes compared to control H3K27me3 domains as demonstrated by the Hi-C analysis. Importantly, a comparison of the MRRs with the ReSE-identified silencers revealed a significant overlap. The deletion of the two MRRs linked to the *IGF2* and *FGF18* genes demonstrated that they indeed acted as strong silencers (Cai et al., 2021). Enrichment in the Polycomb repressive complex 2 (PRC2), which deposits H3K27me3, also underlined a search for silencers in mouse embryonic stem cells (ESCs). To identify chromatin loops associated with PRC2, the chromatin was cross-linked, and genomic regions connected by PRC2 were captured by proximity ligation followed by ChIP using antibodies against PRC2 subunits. The analysis revealed that the PRC2-bound promoter-silencer loops are associated with repressed genes. Deletion of selected candidate silencers led to derepression of their target genes *in vitro* and *in vivo*. Interestingly, the authors also observed that the silencers identified in mESCs transition during development into tissue-specific enhancers as the cells differentiate (Ngan et al., 2020).

While the majority of screens for silencers were performed in mammalian cell lines, parallel studies were reported in *Drosophila*. The authors selected ∼600 candidate regions based on their DNase I hypersensitivity, H3K27me3 enrichment, and binding of Groucho and CtBP co-repressors, and then screened them using a GFP reporter in *D. melanogaster* embryos. Interestingly, nearly all the identified silencers were demonstrated to act as active enhancers in other cellular contexts, challenging the rigid separation of regulatory elements into silencers and enhancers (Gisselbrecht et al., 2020). Even more striking findings were reported recently by Hofbauer et al. (2024). Employing a genome-wide (randomly sheared entire *Drosophila* genome) high-throughput reporter assay screen in S2 cells, they identified 837 silencers. Intriguingly, the silencers do not exhibit open chromatin, largely lack histone modifications, and are enriched in the Phaser, Suppressor of Hairy-wing (Su(Hw)), and *Drosophila* long motif 3 (DLM3) DNA motifs. Depletion of each of the corresponding TFs led to the loss of silencing of their respective target genes. For instance, the DLM3 motif is recognized and bound by Saft, a zinc-finger TF that interacts with the G9a histone methyltransferase (independent of its H3K9-methyltransferase activity) and represses tracheal and cuticle gene programs in non-tracheal/epidermal tissues. This study demonstrated that silencers also operate beyond the distinct epigenetic signatures and emphasized the importance of unbiased screens (Figure 2C) (Hofbauer et al., 2024). It also highlighted the need for context-specific *in vivo* testing of candidate silencers, similar to that required for enhancers. Analogously to SEs, super-silencers (SSs) have recently been reported. The *FGF18* gene is linked to two silencer elements, enriched in H3K27me3, which cooperate as an SS through compensatory chromatin interactions to synergistically repress the *FGF18* gene. Compared to single knockouts (KOs), the double KO of both silencers led to synergistic upregulation of *FGF18*, resulting in gene expression changes and shifts in cell identity (Zhang et al., 2025). It is very likely that further studies will identify more SS-regulating developmental genes in various cellular contexts. Taken together, unlike enhancers, silencers often lack a characteristic molecular signature, but like enhancers, act in a highly context-dependent manner, as bifunctional repressive or activating cis-regulatory elements.

## Enhancers in human trophoblast

Although the placenta is a vital organ that supports fetal growth and development, the transcriptional regulatory mechanisms governing its formation and function remain poorly understood. In particular, the processes by which distinct trophoblast cell lineages and types are specified are not well defined. A key open question is how enhancer hubs integrate chromatin states, TFs, signaling pathways, and the general transcriptional machinery to spatiotemporally orchestrate context-specific gene expression programs during placental development (Papuchova and Latos, 2022). Recent advances, particularly the emergence of high-resolution omics technologies and the establishment of robust *in vitro* trophoblast models, have begun to shed light on these complex regulatory networks. Experimental approaches typically involve loss- and gain-of-function studies, combined with transcriptomic profiling and the identification of TF chromatin binding sites and associated chromatin states. TSC and organoid models, supported by reference data from primary placental tissues, offer powerful systems to investigate TF roles not only in CTB progenitors, but also during their differentiation to STB and EVT (Table 2) (Baczyk et al., 2009; Hubert et al., 2010; Renaud et al., 2014; 2015; Liu et al., 2015; Krendl et al., 2017; Haider et al., 2018; Lv et al., 2019; Dong et al., 2020; Dong et al., 2022; Saha et al., 2020; Hornbachner et al., 2021; Varberg et al., 2021; Varberg et al., 2023; Chen et al., 2022; Jeyarajah et al., 2022; Karvas et al., 2022; Ray et al., 2022; Dietrich et al., 2023; Frost et al., 2023; Kim et al., 2023; Shimizu et al., 2023; Yu et al., 2023; Esbin et al., 2024; Wang et al., 2024; Yi et al., 2024; Zheng et al., 2024; Guo et al., 2025). Similarly, these models were used to demonstrate that chromatin rewiring at regulatory elements is essential for trophoblast differentiation. Depletion or pharmacological inhibition of the histone acetyltransferase P300, histone demethylase LSD1, and histone deacetylases HDAC1/2 impaired STB and EVT formation, highlighting their critical role -likely via enhancer regulation-in trophoblast lineage commitment (Milano-Foster et al., 2019; Jaju Bhattad et al., 2020; van Voorden et al., 2023).

**TABLE 2:** Summary of *cis*-regulatory elements, TFs, and their depletion effects in trophoblast.

Lineage	Regulatory elements	Key TFs	Loss-of-function TF phenotype		Study
CTB progenitors	Enhancers, SEs (p300, H3K27ac)	TEAD1	impaired proliferation and SR in TSCs		Chen et al. (2022), Dong et al. (2022), Kim et al. (2023)
		TEAD4	reduced proliferation and SR in TSCs, premature differentiation bias to STB (*CGB* and *ERVW-1* upregulated)		Chen et al. (2022), Saha et al. (2020), Ghosh et al. (2024)
		GATA2	slightly reduced SR in TSCs (GATA3 compensation), upon differentiation failure to form STB (STB markers downregulated)		Krendl et al. (2017), Chen et al. (2022), Ghosh et al. (2024)
		GATA3	loss of SR, clonogenicity, reduced proliferation in TSCs, premature differentiation bias to STB		
		TFAP2C	loss of SR, downregulation of cell cycle markers, reduced clonogenicity in TSCs		Dong et al. (2022), Chen et al. (2022), Kim et al. (2023)
		FOS	reduced proliferation and clonogenicity, increased expression of EVT invasiveness markers (*MMPs* and *CDH11*) in TSCs		Renaud et al. (2014), Dong et al. (2022)
		MAFK	impaired proliferation and SR maintenance, decreased clonogenicity in TSCs		Dong et al. (2022)
		MSX2	loss of SR and reduced proliferation in TSCs, spontaneous differentiation to STB (*CGB*, *CGA*, *PSG(s)* upregulated)		Hornbachner et al. (2021)
		PPARG	downregulation of SR genes, reduced clonogenicity and proliferation in TSCs, defective induction of EVT differentiation		Guo et al. (2025)
		NR2F2/MAZ	significantly decreased SR and reduced clonogenicity in TSCs		Hubert et al. (2010), Chen et al. (2022)
EVT/STB differentiation	ERV/LTR-derived (LTR10A, MER41B, MER50) (H3K27ac, H3K4me1)	TFAP2C	loss of the elements leads to decreased expression of EVT (*MMPs*, *HLA-G*) and STB (*CGB*, *CGA*, *PSG(s)*) genes and binding of these TFs, impaired initiation of STB and EVT differentiation programs		Frost et al. (2023), Yu et al. (2023), Dong et al. (2022)
		TEAD4			
		GATA3			
		JUN			
EVT differentiation	Dynamic enhancers (open chromatin and H3K27ac)	TFAP2C	upon EVT differentiation impaired induction of EVT markers (*HLA-G*, *MMP2*) and reduced invasiveness		Kim et al. (2023)
		DLX5			
		DLX6			
		ZNF439			
		SNAI1	compatible with SR state, upon EVT differentiation failure in elongation and invasiveness, reduced expression of EVT markers (*ITGA1*, *HLA-G*, *MMP2*)		Varberg et al. (2023)
		EPAS1			
		TEAD1	failed to complete EVT differentiation, bias towards STB differentiation program		Dong et al. (2022)
		GCM1	compatible with SR state, upon EVT differentiation failed induction of EVT program, and migration (WNT signaling reduced)		Shimizu et al. (2023), Varberg et al. (2021)
		DLX3			
		ASCL2	blocking of EVT differentiation, promotion of STB differentiation		Jeyarajah et al. (2022), Varberg et al. (2021), Varberg et al. (2023)
		PPARG	upon EVT differentiation impaired invasion ability, decreased expression of EVT markers		Guo et al. (2025)
		RXRA			
STB differentiation	ERV-derived enhancers, enhancers	GCM1	upon differentiation to STB failed induction of STB differentiation program, decreased secretion of CGB, loss of cell fusion		Baczyk et al. (2009), Jeyarajah et al. (2022), Wang et al. (2024)
		DXL3			
		OVOL1	impaired STB transcriptional program and syncytialization, decreased expression of ERVs and hormone production		Renaud et al. (2015), Shimizu et al. (2023)
		TFEB	abrogation of STB formation and failed syncytialization, decreased expression of STB markers (*CGB*, *PSG(s)*, *ERVs*)		Esbin et al. (2024), Zheng et al. (2024)
		TBX3	impediment of STB formation and failed syncytialization through reduced RAS-MAPK signaling		Lv et al. (2019), Yi et al. (2024)
		STAT5A/MITF	required for STB differentiation through co-operation with *PAPPA* and *FOSL2*		Wang et al. (2024)
mTSCs self-renewal	SEs (p300, H3K27ac, Med12, open chromatin)	Sox2	Embryonic lethality / placental phenotype	downregulation of cell cycle and SR genes, upregulation of differentiation markers	Adachi et al. (2013)
		Esrrb		downregulation of key TS-specific TFs leading to differentiation	Latos et al. (2015b), Gao et al. (2019)
		Eomes		downregulation of TSC markers, upregulation of TGS and SynT markers	Russ et al. (2000), Latos et al. (2015a)
		Cdx2		downregulation of TSC markers, loss of stemness and proliferation	Strumpf et al. (2005), Rayon et al. (2016), Bisia et al. (2025)
		Elf5		skewed balance away from SR towards differentiation, downregulation of *Tfap2c*, *Eomes*, and *Cdx2*	Donninson et al. (2005), Latos et al. (2015a)
		Tfap2c		loss of SR, differentiation towards SpT and TGCs, downregulation of *Cdx2*, *Eomes*, and *Elf5*	Kuckenberg et al. (2010), Lee et al. (2019)
		Tead4		destabilization of SR network (*Eomes*, *Elf5*, *Cdx2* downregulated), skewed differentiation upon induction	Yagi et al. (2007), Murphy et al. (2024)
		Zfp281		loss of SR (*Elf5*, *Cdx2* downregulated), skewed differentiation upon induction	Ishichui et al. (2019)
		Ets2		loss of SR (*Elf5*, *Cdx2*, and *Tfap2c* downregulated), reduced proliferation	Polydorou and Georgiades, (2013)
mTSCs differentiation	SEs decommissioning (p300, H3K27ac)	Erf-NCoR1/2	*Erf* dispensable in SR, attenuated exit from SR leading to delayed differentiation		Papadaki et al. (2007), Lackner et al. (2023)
		Mafk	upon differentiation suppression of expression of invasive SpTs (*Tpbpa*) and spiral artery TGC markers (*Prl2c2* and *Fosl1*)		Polydorou and Georgiades, (2013), Lee et al. (2019)
		Foxj2			
		Ets2			
		Hopx	upon differentiation enhanced expression of spiral artery TGC markers (*Prl2c2* and *Fosl1*)		
		Pou3f1	upon differentiation enhanced expression of invasive SpT (*Tpbpa*) and spiral artery TGC (*Prl2c2* and *Fosl1*) markers		
		Meisl			
		Zfpm1			
		Tfeb	disabled STB formation		Zheng et al. (2024)

Summary of studies on *cis*-regulatory elements controlling trophoblast development in human and mouse, including associated epigenetic signatures, TF binding, and functional evidence from TF knockdown or knockout experiments.

### TFs and enhancers sustaining CTB

To gain insights into the transcriptional regulation of CTB, Kim et al. identified enhancers in the hTSCs (Kim et al., 2023). Based on the chromatin occupancy of the histone acetyltransferase P300 — a key enzyme associated with the deposition of the active enhancer mark H3K27ac—they identified 31,362 putative enhancers, predominantly located in distal genomic regions. These enhancers were enriched for binding motifs of the prominent trophoblast TFs, including TEAD4, GATA2/3, and TFAP2C. Using the ROSE algorithm, the authors identified 549 SE-associated genes, among them 76 TFs. These SE-associated genes encompassed core regulators of the TSC state (e.g., TEAD4, GATA2/3, TFAP2C, FOS, and MAFK) as well as TFs with previously uncharacterized roles in trophoblast biology (e.g., NCOR2, RCOR1, and ZNF362). Chromatin profiling of the 5 selected TFs (FOS, TEAD4, TFAP2C, GATA2, and MAFK) revealed that these factors frequently co-occupied regulatory regions of target genes, forming a gene regulatory network (GRN) characterized by autoregulatory loops, feed-forward interactions, and extensive interconnectivity. To further dissect this network, the authors categorized regulatory regions into five groups based on the number of TFs co-bound. Analysis revealed that gene expression levels increased with the number of co-occupying TFs, indicating that TSC core TFs bind to the enhancers in a combinatorial manner and act collaboratively to drive the progenitor programs. In contrast, genes bound by only one or two of these TFs were expressed at low levels in TSCs and were predominantly associated with differentiation programs. Functional depletion experiments revealed that all 5 TFs exhibit dual functionality, acting as both activators and repressors. While FOS, MAFK, and TEAD4 tend to activate their target genes, TFAP2C showed a slight bias toward repression. Together, these findings suggest that FOS, MAFK, TEAD4, GATA2, and TFAP2C form a synergistic transcriptional circuit that sustains the TSC state by activating self-renewal genes and repressing differentiation-associated programs (Kim et al., 2023).

Placental morphology and development display wide variation across species, and it is thought that the highly species-specific transposable elements (TEs), in particular the endogenous retroviruses (ERVs), are the major drivers of the fast evolution of this organ. The most prominent example of the TE-derived genes is syncytins, which are essential for cell-cell fusion and formation of the multinucleated syncytiotrophoblast layer. An important role is also played by the non-coding portions of TEs, like the long terminal repeats (LTRs). They have been shown to act as promoters (e.g., LTR10A (*NOS3* gene), LTR2B (*PTN* gene), MER39 (*PRL* gene), MER39B (*ENTPD1* gene), MER21A (*HSD17B1* and *CYP19A1* genes) and enhancers (Cohen et al., 2009; Frost et al., 2023). Recent analysis in CTB and human TSCs identified ERV-associated LTRs exhibiting hallmarks of regulatory activity, including high levels of H3K27ac, H3K4me1, and binding by TFs, including GATA3, TEAD4, TFAP2C, JUN, and JUND (Frost et al., 2023). Functional validation using CRISPR excision of candidate regions revealed that expression of placental genes: *CSF1R* (colony stimulating factor 1 receptor; promotes growth, proliferation, and migration of trophoblast), *PSG5* (pregnancy specific glycoprotein 5, specifically expressed by STB and EVT), and *ENG* (endoglin; contributes to and is marker of preeclampsia) is controlled by the neighboring LTR elements acting as enhancers (LTR10A, LTR8B, and MER41B, respectively). Similarly, the ERV-derived enhancers play a key role in trophoblast syncytialization. Yu et al. identified MER50 ERV-derived bivalent, H3K27ac and H3K9me3 enriched, enhancers associated with STB genes in hTSCs. During STB differentiation, the bivalent enhancers gain H3K27ac, lose H3K9me3, get activated, and drive expression of the adjacent STB genes, including *MFSD2A* and *TNFAIP2*. Deletion of these elements attenuates expression of associated genes, resulting in compromised STB differentiation (Yu et al., 2023). In summary, these findings demonstrate the importance of transposable elements in trophoblast gene regulation and placental evolution.

While comprehensive studies of enhancers are essential, experiments that combine chromatin binding analysis with functional depletion of a single TF have also yielded invaluable insights into the GRN operating in undifferentiated trophoblast (Table 2). Recent studies have revealed that TEAD4 (along with its co-activators YAP1 and WWTR1), GATA2, GATA3, MSX2, TFAP2C, PPARG, ΔNp63α are pivotal regulators of the progenitor state, driving proliferation, cell cycle progression, and expression of the stemness genes (Meinhardt et al., 2016; Saha et al., 2020; Hornbachner et al., 2021; Varberg et al., 2021; Ray et al., 2022; Wang et al., 2022; Ghosh et al., 2024; Guo et al., 2025). Importantly, some of these factors simultaneously repress differentiation programs. For example, depletion of TEAD4, YAP1, WWTR1, and MSX2 results in a loss of self-renewal, activation of STB programs, and differentiation into STB cells. Chromatin binding analysis revealed that these TFs occupy not only the active genes associated with stemness, but also the STB-specific genes (e.g., CG and PSG gene families) that are not expressed in the CTB context. These findings suggest that such TFs may function as both activators and repressors while binding the cis-regulatory regions bearing enhancer signatures. Thus, the underlying mechanism rather relies on temporarily “incapacitating” an enhancer than binding to a silencer and may reflect the requirement of seamless differentiation. Indeed, Hornbachner et al. demonstrated that depletion of MSX2 TF in TSCs resulted in activation of the STB program, while forced expression blocked it. A large proportion of the affected genes were co-bound by MSX2 and the cBAF chromatin remodeling complex. Increased H3K27ac and cBAF occupancy upon MSX2 depletion implies that MSX2 prevents premature syncytiotrophoblast differentiation (Hornbachner et al., 2021).

Interestingly, several TFs operate in both undifferentiated and differentiated trophoblast. For example, TEAD1, TFAP2C, and SNAI1 are essential for both the CTB progenitor state and differentiation toward EVT, whereas GATA2 and GATA3 are critical for both the CTB progenitor state and STB formation (Ghosh et al., 2024). To further elucidate the mechanisms underlying these multifaceted roles, it will be important to investigate the dynamics of enhancer function and TF binding. To understand these processes, it would be interesting to follow enhancer activity and TF chromatin binding patterns and integrate them with signaling inputs throughout trophoblast differentiation.

### TFs and enhancers operating in EVT

**FIGURE 3:**
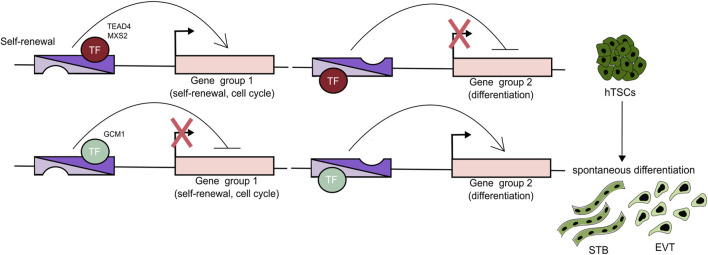
TFs can act as activators and repressors. A Trophoblast cell fate decisions are orchestrated by enhancers that regulate the expression of genes specific to different trophoblast lineages. These enhancers are bound by TFs that function as both activators and repressors. For example, TEAD4 and MSX2 are key regulators of the progenitor state, promoting proliferation and the expression of stemness-associated genes while concurrently suppressing differentiation pathways. In contrast, TFs such as GCM1 promote differentiation by activating programs associated with both STB and EVT lineages, while inhibiting self-renewal and progenitor-specific gene expression.

Trophoblast differentiation is characterized by the global, dynamic chromatin and transcriptional changes. Two recent studies followed this transformation during the EVT differentiation. By integrating a time-course of chromatin accessibility, long-range chromatin interactions, transcriptomics, and TF binding motif enrichment, Varberg et al. identified key EVT enhancers and uncovered TFAP2C, SNAI1, and EPAS1 as essential regulators of the EVT differentiation (Varberg et al., 2023). Depletion of TFAP2C affected both the stem cell state and the early stages of EVT differentiation, whereas depletion of SNAI1 and EPAS1 was compatible with the TSC state, but adversely impacted EVT differentiation outcomes. Further analysis revealed that SNAI1 and EPAS1 act predominantly as repressors. TFAP2C was further validated as a major EVT regulator by an alternative approach (Kim et al., 2023). Based on four distinct expression patterns during TSC to EVT differentiation, Kim et al. classified the genes into four classes (C1: high in TSCs, downregulated upon differentiation, C2: inactive in TSCs, upregulated upon differentiation, C3: high in early, down in late differentiation, C4: high in TSCs and EVTs, downregulated in between) and mapped the corresponding changes in enhancer landscape using the H3K27ac, P300, MED1, and H3K4me1 hallmarks. The analysis revealed 7 SE-associated TFs (DLX5, DKLX6, ASCL2, ZNF439, IRF7, SNAI1, and VAX2) in class 2 and 39 in class 3, including TFAP2C, whose motif was overrepresented in enhancers. Functional validation of these candidates demonstrated that the early EVT differentiation is driven by TFAP2C acting as a pioneer factor, and priming multiple EVT genes, including the late-stage TFs. The late-stage TFs, in turn, act together as a network that activates and executes the EVT-differentiation program. Several additional studies, including CRISPR screens, have provided further insights into the TFs driving EVT differentiation, identifying ASCL2, GCM1, TEAD1, PPARG, and DLX3 as key regulators. Functional depletion experiments, combined with chromatin profiling, revealed that these TFs bind to enhancers and activate EVT expression programs (Dong et al., 2022; Shimizu et al., 2023; Varberg et al., 2023; Guo et al., 2025). Notably, depletion of GCM1 and DLX3 disrupted differentiation into both EVT and STB, indicating their broader role in trophoblast development. In contrast, disruption of ASCL2 and TEAD1 impaired EVT while simultaneously promoting STB fate. These findings suggest that ASCL2 and TEAD1 may act as dual-function TFs-supporting EVT specification while repressing alternative lineage programs such as STB. Together, EVT differentiation involves dynamic chromatin and transcriptional remodeling driven by TFs (e.g., TFAP2C, SNAI1, EPAS1, ASCL2, GCM1, TEAD1, PPARG, and DLX3), which regulate enhancer activity and expression of lineage-specific genes, while balancing alternative trophoblast fates (Figure 3) (Varberg et al., 2023). Future studies are likely to uncover additional regulatory networks, pioneer factors, and non-coding elements that fine-tune lineage decisions, potentially offering therapeutic insights into placental disorders such as preeclampsia.

### TFs and enhancers operating in STB

While the TFs and chromatin landscapes associated with STB have been relatively well characterized, the cis-regulatory elements, particularly enhancers, remain less understood. Our initial understanding of the TFs driving STB formation comes from experiments using primary CTB, which spontaneously differentiate into STB, and choriocarcinoma cell lines. Recent loss-of-function studies, including CRISPR-Cas9–based genetic screens in differentiating TSCs, have revealed both established (e.g., GCM1, OVOL1, DLX3) and novel (e.g., TFEB, GRHL1, CEBPA) regulators of STB differentiation. GCM1 functions as a master regulator of STB in both human and mouse placentae. Functional depletion of either GCM1 or DLX3 prevents STB (and EVT) differentiation, while forced expression of GCM1 promotes these fates (Jeyarajah et al., 2022; Shimizu et al., 2023). GCM1 and DLX3 bind to regulatory regions, including promoters and enhancers, to activate transcription of STB-specific genes, notably placental hormones and the fusogenic proteins SYNCYTIN-1 and -2, which are encoded by the endogenous retroviruses HERV-W and HERV-FDR, respectively. These proteins are essential for trophoblast cell fusion and the formation of the multinucleated syncytiotrophoblast layer. Additional TFs such as OVOL1, PPARG, TBX3, and TFEB have also been implicated in STB lineage commitment. TFEB has emerged as an STB-specific regulator, with no apparent role in EVT differentiation (Lv et al., 2019; Esbin et al., 2024; Yi et al., 2024; Zheng et al., 2024). TFEB depletion abrogates STB formation and syncytialization, while overexpression enhances these processes. TFEB directly activates ERVFRD-1 (SYNCYTIN-2) expression without affecting GCM1, and GCM1 loss does not alter TFEB, indicating that these TFs operate independently.

To further dissect the regulatory logic of STB development, recent work by Wang and colleagues applied integrated single-nucleus (sn)RNA-seq and snATAC-seq on STBs from early and late gestation placentas (Wang et al., 2024). This approach revealed substantial heterogeneity within STB nuclei and identified a bifurcated developmental trajectory originating from a common progenitor toward two mature STB subpopulations: eSTB mature 1 and eSTB mature 2, connected by a transitional intermediate state. By integrating gene expression and chromatin accessibility profiles into enhancer–gene regulatory networks (eGRNs), the study identified putative master regulators of these STB subtypes. ZNF217, RELA, MAFK, and GRHL3 were associated with early CTB fusion; STAT5A, STAT5B, STAT4, and MITF with eSTB mature 1; and FOSL2, CEBPB, CEBPG, and SP3 with eSTB mature 2. For instance, MITF-associated enhancers were linked to hormone genes, including *CSH2* and *LEP*, suggesting that MITF plays a role in placental endocrine function. Functional validation by doxycycline-inducible expression of *STAT5A* and *MITF* in TSCs, combined expression and ChIP-seq analysis confirmed direct binding of these TFs to target enhancers within the previously identified eGRNs, providing functional evidence for their role in STB lineage specification. Collectively, these findings highlight the complex, multi-layered transcriptional and enhancer-based regulatory programs that govern STB differentiation. Future studies should aim to functionally characterize these enhancers and delineate the hierarchical TF networks that regulate their activity across developmental time points and trophoblast subtypes.

## Enhancers in murine trophoblast

The progenitor identity of the mouse ExE *in vivo* and TSCs *in vitro* relies on Fgf signaling, driving a distinct network of TFs. Sox2 and Esrrb TF are the critical upstream members of this network, as their ablation causes placental embryonic lethality and differentiation of TSCs, while their constitutive overexpression confers Fgf-independent self-renewal and multipotency (Adachi et al., 2013; Latos et al., 2015b). The network involves Tfap2c, Tead4, Gata2/3, Elf5, Eomes, Cdx2, Zfp281, among other TFs. They bind a wide range of target genes, including each other’s and their own enhancers, and drive their expression, sustaining self-renewal and multipotency of mTSCs (Table 2) (Russ et al., 2000; Donnison et al., 2005; Strumpf et al., 2005; Papadaki et al., 2007; Yagi et al., 2007; Kuckenberg et al., 2010; Adachi et al., 2013; Polydorou and Georgiades, 2013; Latos et al., 2015a; Latos et al., 2015b; Rayon et al., 2016; Gao et al., 2019; Ishiuchi et al., 2019; Lee et al., 2019; Lackner et al., 2023; Murphy et al., 2024; Zheng et al., 2024; Bisia et al., 2025). For instance, Esrrb binds itself as well as Elf5 and Eomes, and is essential for their expression (Latos et al., 2015b). Interestingly, the relative abundance of Elf5, Eomes, and Tfap2c regulates trophoblast differentiation. While in TSCs, Elf5, Eomes, and Tfap2c co-occupy and drive expression of the TSC-specific genes, at the onset of differentiation, the changes in abundance of these TFs result in a shift in target genes of Tfap2c and Elf5, resulting in activation of genes promoting differentiation (Latos et al., 2015a).

TFs often interact and collaborate with chromatin-modifying and -remodeling complexes. For example, Eomes has recently been shown to associate with the canonical BAF (cBAF) complex, a member of the SWI/SNF family of ATP-dependent chromatin remodeling complexes (Bisia et al., 2025). These complexes regulate chromatin accessibility by repositioning nucleosomes, thereby modulating gene expression. Eomes, together with Tfap2c, Essrb, Zfp281, Oct6, and Klf5, functions as part of a GRN and recruits the cBAF to enhancers of key trophoblast genes. This ensures chromatin accessibility and gene expression, thereby maintaining the TSC state (Bisia et al., 2025).

One of the first attempts to comprehensively characterize enhancers in murine trophoblast was undertaken by Lee et al. in TSCs (Lee et al., 2019). The enhancers (and the SE) were mapped based on p300 occupancy and validated by enrichment in H3K27ac, H3K4me1, and Med12, and exhibited prominent trophoblast specificity (over 75% compared to ESCs). Interestingly, expression of TFs (e.g., Esrrb, Trim71, Tead1) in TSCs and ESCs was regulated by a distinct set of enhancers in each lineage, suggesting enhancer specificity. Considering the strength of the p300/H3K27ac/Med12 signal combined with the chromatin accessibility assessed by ATAC-seq, the authors identified 1046 SE-associated genes, enriched in the placenta-related GO terms. Among them, 197 SE were associated with TFs, epigenetic regulators, and/or DNA-binding proteins, and included well-established trophoblast regulators Esrrb, Cdx2, Elf5, Gata3, Hand1, Sox2, Tfap2c, and Tead4. The ChIP-seq analysis of the selected 28 SE-associated TFs, both known (Elf5, Eomes, Tfap2c, Tead4, Hopx, Ets2, cFos, Arid3a, Id2, Mef2d) and unknown (Pcgf5, Smad6, Lrrfip1, Pou3f1, Cbfa2t3, Creb3l2, Bbx, Tbx20, Mafk, Maff, Fbxo21, Zfpm1, Irf2, Hic2, Foxj2, Bhlhe40, Meis1) and Ctcf revealed a highly intertwined TSC-specific transcriptional regulatory network (TRN) operating in TSCs (Lee et al., 2019). The newly defined TFs not only regulated the known TFs but also co-occupied a shared set of target genes (1,296 target genes co-occupied by > 22 TFs) and worked together to promote trophoblast gene expression. Interestingly, many TFs within the network regulate each other collaboratively rather than hierarchically, and most of the TFs in the TRN are regulated by feed-forward, feedback, and auto-regulatory mechanisms. Additional insights into the 3D enhancer connectivity in mTSC were recently provided by Murphy et al. (2024).

Differentiation of TSCs is associated with global transcriptional changes driven by enhancers. Accordingly, a comparison of enhancer usage revealed the emergence of enhancers linked to differentiation genes in conjunction with the loss of enhancers linked to the TSC self-renewal and multipotency programs, indicating dynamic changes in the enhancer landscape that occur during differentiation and, more broadly, during placental development. In addition, depletion experiments followed by TSC differentiation have demonstrated that while Maff, Mafk, Foxj2, or Ets2 KD resulted in impaired induction of the invasive SpTs and spiral artery TGC, the Meisl, Pou3f1, Id2, Pcgf5, Hopx, or Zfpm1 KD led to the opposite effect, i.e., stronger activation of these programs (Table 2) (Lee et al., 2019). Together, this pioneering study expanded our understanding of the TRN operating in the trophoblast lineage and placental development.

Interesting insights into how the signaling cues are transformed into repressive mechanisms operating at the onset of the TSC differentiation were recently provided by Lackner et al. (2023). They identified Erf as a direct phosphorylation target of Fgf/Erk and demonstrated that upon attenuation of this pathway in TSCs, the unphosphorylated Erf translocates to the nucleus and recruits the Nuclear Co-Repressor (NCoR) 1/2 complex to the trophoblast genes. Mechanistically, Erf brings about transcriptional silencing of these genes by recruitment of the NCoR1/2 complex to the SEs associated with the key trophoblast regulators (including *Esrrb*, *Elf5*, *Eomes*, *Cdx2*, *Tead4*, *Sox2*) and decommissioning them, including by histone deacetylation carried out by the NCoR1/2 subunit Hdac3. Genetic ablation of either Erf or Tbl1x (a component of the NCoR1/2 complex) abrogated the Erf-NCoR1/2 interaction and resulted in a severe TSC differentiation defect due to the faulty SE decommissioning, lack of silencing, and persistent expression of the self-renewal marks associated with these SEs (Lackner et al., 2023). This study yet again demonstrated that developmental cell fate transitions are controlled by the signaling inputs, integrated at the enhancer level, and transformed into specific gene expression patterns.

As in humans, murine transposable elements and endogenous retroviruses also contribute to the placental enhancer landscape. Studies in mouse trophoblast revealed that the long terminal repeat families LTR, in particular RLTR13B and RLTR13D5 display tissue-specific hallmarks of enhancers, including accessible chromatin, high levels of H3K27ac, enrichment in binding motifs for trophoblast TFs Elf5, Cdx2, Eomes and in proteins associated with enhancers including p300, Mediator complex, cohesins (Chuong et al., 2013; Todd et al., 2019). Interestingly, functional genetic validation experiments revealed that only a subset of these regions acts as true enhancers. For instance, the deletion of chr18-RLTR13B4 and chr16-RLTR13D5 enhancers, associated with the *Map3k8* and *Scarf2* genes, respectively, resulted in a substantial reduction of their expression in TSCs (Todd et al., 2019). Together, these findings demonstrate co-option of transposable elements as trophoblast-specific enhancers.

## Summary and outlook

Recent advances have significantly deepened our understanding of the TF networks and enhancer landscapes that govern human trophoblast development. High-throughput technologies, including CRISPR-based functional screens and multi-omics profiling, have enabled the identification of key TFs and cis-regulatory elements driving lineage-specific programs in CTB, STB, and EVT. Moving forward, integrative approaches will be essential to reconstruct the full regulatory architecture of trophoblast lineages. Additionally, attention should expand beyond TFs to include chromatin remodelers, modifiers, coactivators, and corepressors—components that have been relatively neglected yet are crucial for maintaining lineage fidelity by repressing alternative fates. Future studies should also prioritize systematic screens for cis-regulatory elements, including enhancers and, in particular, silencers. Given the specialization of trophoblast lineages, transcriptional repression likely plays an underappreciated role in establishing and maintaining cell identity. Importantly, emerging evidence challenges the traditional binary view of enhancers and silencers, highlighting the need for a more nuanced understanding of cis-regulatory logic. In the coming years, comprehensive, systems-level analyses will be key to fully elucidating the regulatory networks that control trophoblast development, offering critical insights into human placental biology and its associated disorders.
